# Membrane-Spanning Nanopores Formed from Nucleic Acids

**DOI:** 10.1021/acs.chemrev.5c00905

**Published:** 2026-05-22

**Authors:** Yunxuan Li, Tim Karrasch, Ulrich F. Keyser, Kerstin Göpfrich

**Affiliations:** † Cavendish Laboratory, 2152 University of Cambridge, 19, J J Thomson Avenue, Cambridge CB3 0HE, United Kingdom; ‡ Center for Molecular Biology of Heidelberg University (ZMBH), 9144 Heidelberg University, Berliner Straße 45, 69120 Heidelberg, Germany; ¶ Max Planck School Matter to Life, Jahnstraße 29, 69120 Heidelberg, Germany

## Abstract

Transmembrane-spanning nanopores have emerged as powerful tools in a wide range of technological applications, particularly in single-molecule sensing. This review explores recent advancements in creating synthetic, membrane-spanning nanopores constructed from nucleic acids, focusing on DNA nanopores. These self-assembled nanochannels offer a highly programmable and versatile alternative to traditional protein-based nanopores. We summarize the rational design principles and examine advantages and disadvantages of diverse architectures ranging from subnanometer channels for selective ion translocation to customizable geometries for the transport of macromolecules. Key aspects of this emerging field are discussed, including methods for membrane anchoring, the influence of lipid rearrangements on ionic conductance, and the dynamic control of nanopore function. Nucleic acid nanopores are further highlighted as functional components for synthetic cell signaling, single-molecule detection, and cellular manipulation. This review concludes with an outlook on the field, focusing in particular on the unique opportunities of RNA origami for creating genetically encodable nanopores for bottom-up synthetic biology.

## Graphical abstract

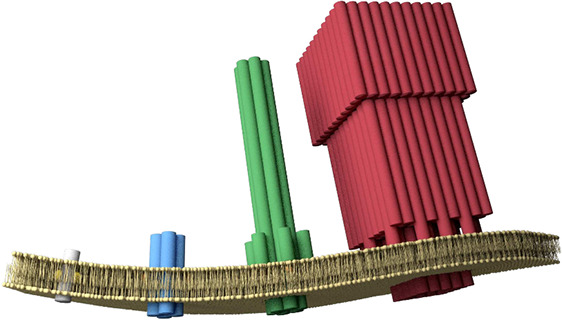

## 1 Introduction

The emergence of nucleic acid nanotechnology—building with DNA and RNA molecules—has revolutionized the field of nanoscale engineering. Among numerous innovations, self-assembled nanopores made from nucleic acids instead of proteins represent a particularly fascinating development. These synthetic nanochannels, precisely engineered in terms of size, conductance properties and gating, have opened new avenues in biosensing, molecular transport, synthetic cell signaling and cellular applications.

Generally speaking, DNA nanopores can be broadly classified into two categories based on their assembly strategies: scaffolded DNA origami nanopores and scaffold-free DNA tile/brick nanopores.^1^ DNA origami structures^2–4^ are constructed by folding a long single-stranded scaffold through hybridization with hundreds of synthetic short oligonucleotide (oligo) staples, conferring high structural stability and mechanical robustness. They commonly exhibit molecular weights on the megadalton (MDa) scale, often around 5 MDa when using the standard M13 scaffold. In contrast, unscaffolded tile/brick structures^5–7^ are assembled from a small number of short oligos (typically 8–16 strands, each around 60 nucleotides, nt). The total molecular weight of such assemblies is on the order of hundred kDa (e.g., 12 strands × 50 nt × 330 Da/nt), making them an order of magnitude smaller than their scaffolded counterpart.

Despite the different assembly approaches, a common architectural feature of both DNA nanopore types is the presence of transmembrane-spanning DNA helices that are usually oriented perpendicularly to the membrane surface. DNA nanopores interact with lipid bilayers through strategically placed hydrophobic modifications^8–10^ or molecular recognition pairs,^11,12^ which can be distributed either along the length of the transmembrane region,^13–15^ or concentrated in raft-like regions that dock onto the surface of the membrane.^16–18^ The design flexibility, combined with the inherent modularity of DNA nanotechnology, gives DNA nanopores significant advantages over their biological counterparts, protein nanopores, in terms of customization and functional modification.

Over the past decade, researchers have made substantial progress in optimizing synthetic DNA nanopores. Various innovative strategies have emerged to address challenges related to membrane insertion,^14,15^ channel conductance,^17,19^ and molecular selectivity,^12,18^ guided by sophisticated computational modeling,^20,21^ advanced characterization techniques,^22^ and systematic experimental studies.^11^ A comprehensive understanding of the underlying principles governing DNA nanopore design and function is essential for harnessing their full potential in biophysics, molecular diagnostics, and targeted therapeutics.

RNA versions of nanopores^23,24^ are still in their infancy compared to DNA nanopores. This is not surprising as accessible RNA design tools are just beginning to emerge.^25,26^ RNA can take on diverse folds beyond the canonical double helix^27^ and enable molecular recognition through aptamer-target interactions and catalytic activity mediated by ribo zymes. For these reasons, combined with the therapeutic relevance of RNA and its ability to fold during transcription *in vivo* or in cells, we envision that RNA nanopores will soon emerge as an exciting direction in the field.

This review will delve into three key aspects of nucleic acid nanopore technology: (i) pore architectures and design strategies, (ii) interactions between nucleic acids and lipid membranes, membrane anchoring and transport pathways, and (iii) current applications and future perspectives. This review covers original research from 2012, when the first DNA nanopores were realized, until the most recent works from early 2026. We will then give an outlook on how RNA nanostructures could become a new frontier in nanopore design. Through this analysis, we aim to highlight how rational design principles can be leveraged to optimize DNA and RNA nanopores for a wide range of scientific and technological applications.

## 2 DNA Nanopore Architectures and Design Strategies

DNA origami design has become a mature field, with accessible computational tools and experimental procedures. A good summary on design strategies has been written by Wagenbauer et al.^28^ The most common DNA origami design software tool, cadnano, offers a choice of two design geometries, the honeycomb and the square lattice.^29^ The structural variability and precision of DNA nanopores is particularly noteworthy, as they exhibit a remarkable diversity in both size and complexity. These nanopores range from minimalist designs composed of just one DNA duplex^30^ to highly sophisticated assemblies incorporating hundreds of oligos,^16^ with inner diameters spanning from subnanometers^30^ to several tens of nanometers^18,31,32^ (Figure 1a). This hierarchical progression in design complexity not only reflects the evolution of DNA nanotechnology but also broadens the functional scope of DNA nanopores, transforming them from basic “holes in the membrane” into versatile molecular transporters capable of selective transport, biosensing, and molecular recognition. Here, we categorize DNA nanopore designs into three size-based classes—small (<3 nm), medium (4–10 nm), and large ( > 10 nm) defined by their lumen diameter^33^ and discuss their distinct features in the following sections.

**Figure 1:**
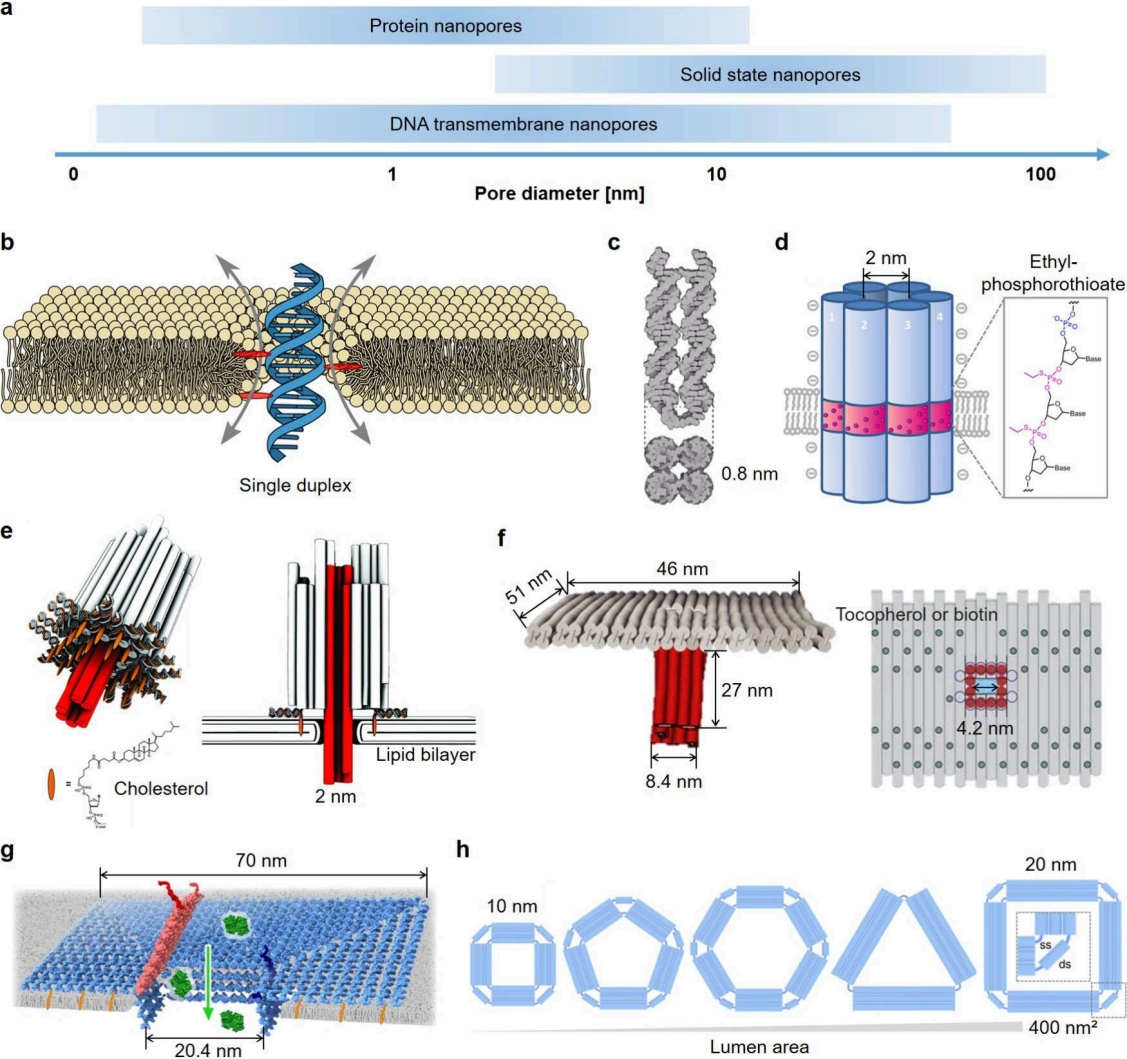
Architectures of membrane-interacting DNA nanopores of various sizes. (a) Typical size ranges of protein nanopores, solid-state nanopores, and DNA transmembrane nanopores. (b-h) Examples of DNA nanopores of increasing sizes. (b) Smallest possible DNA nanopore consisting of a single DNA duplex, revealing lipid reorganization and conductance at the DNA-lipid interface. Reproduced with permission from ref 30. Copyright 2016 American Chemical Society. (c) Square-shaped four helix bundle forming a compact channel with a 0.8 nm diameter. Reproduced with permission from ref 34. Copyright 2022 The Authors. (d) Honeycomb-shaped six-helix bundle forming a channel with a 2 nm diameter, surrounded by an external hydrophobic belt of ethyl-phosphorothioate groups that mask the negative DNA backbone charges for lipid insertion. Reproduced with permission from ref 14. Copyright 2013 American Chemical Society. (e) The first DNA transmembrane pore composed of 54 double-helical domains arranged on a honeycomb lattice (2 nm in inner diameter), with cholesterol-functionalized adaptor strands (orange) facilitating membrane anchoring. Reproduced with permission from ref 16. Copyright 2012 American Association for the Advancement of Science. (f) T-shaped DNA nanopore with a 4.2 nm diameter, consisting of a central stem and a double-layered top plate which provides a large area for membrane interactions. Reproduced with permission from ref 11. Copyright 2016 The Authors. (g) Large gated nanopore with a 20.4 nm × 20.4 nm opening cross-section. Reproduced with permission from ref 18. Copyright 2022 The Authors. (h) Top-down view of DNA nanopore caps with tunable polygonal geometries and lateral dimensions ranging from 10 to 20 nm, covering a lumen area from 100 to 400 nm^2^. Reproduced with permission from ref 31. Copyright 2022 Springer Nature.

### 2.1 Small-Diameter DNA Nanopores (<3 nm)

Small DNA nanopores, typically less than 3 nm in inner diameter, represent an important class of synthetic membrane channels assembled from precisely arranged DNA helices. While comparable in size to or even larger than many biological protein pores, they lie at the lower end of most solid-state nanopores. The fundamental building blocks of these structures are DNA bundle architectures, particularly square-shaped four-helix bundles^34,35^ (4HB) and honeycomb-shaped six-helix bundles^13,14,16^ (6HB), which create central channels with diameters of approximately 0.8 and 2 nm, respectively. Smaller pores offer distinct advantages in terms of ion and molecular selectivity while maintaining effective ion transport.^36^ Scaffold-free helix bundles are easy to design and assemble at micromolar concentrations. Moreover, these nanopores exhibit well-defined conductance values (0.1–2 nS) that closely resemble biological ion channels, enabling biomimetic transport properties and precise molecular detection capabilities.^33^

There are many examples of small DNA nanopores in the literature. The smallest one, a single transmembrane spanning DNA duplex functionalized with porphyrins for membrane insertion, was created to elucidate the conductance pathway of DNA nanopores (Figure 1b).^30^ The duplex was designed such that the three hydrophobic porphyrins can only incorporate into the membrane when the DNA duplex is inserted into the membrane in a perpendicular orientation. Despite the lack of a central pore lumen, insertion steps of 80 pS conductance were observed caused by ion flux at the DNA-lipid interface (see Section 3).

The scaffolded DNA origami technique^2^ has further expanded the possibilities for DNA nanopore design, enabling a broader variety of shapes and a higher number of hydrophobic anchors. A pioneering design, in fact the first membrane-spanning DNA pore, was demonstrated by Langecker et al.^16^ who created a biomimetic nanopore inspired by the shape of α-hemolysin. Their design featured a barrel-shaped cap with 26 cholesterol moieties for membrane adhesion and a six-helical stem forming a 2 nm inner diameter channel (Figure 1e). Electrophysiological measurements revealed that these DNA nanochannels exhibited well-defined conductance and gating responses reminiscent of natural ion channels. Moreover, these nanopores demonstrated excellent capability in discriminating single DNA molecules, enabling the detection of individual oligos passing through the channel.

All-atom molecular dynamics (MD) simulations later predicted fluctuations in the opening diameter at both ends of a 6HB channel on the nanosecond time scale.^21^ These simulations suggested that the channel, with an initial diameter of 2 nm, could transiently constrict to 1.1 nm driven by thermal fluctuations and lipid rearrangements—narrower than the 1.2 nm threshold required for hydrated single-stranded DNA (ssDNA) passage. The kinetics of these conformational changes generated characteristic signal-like traces indicative of nanopore gating transitions. Furthermore, the 6HB channel was observed to exhibit voltage- and conformation-dependent conductance switching between two distinct states.^16^ It preferentially adopted a low-conductance state at high voltages, especially when incorporated into a nanopipette-mounted bilayer under mild negative pressure.

Small nanopores can, in principle, also be constructed from RNA. While RNA origami has not yet achieved structures as large as the typical scaffolded DNA origami, structures of up to 2500 nucleotides^25^ have been achieved cotranscriptionally and could, in principle, enclose a pore cavity. Cryo electron microscopy has shown that RNA nanostructures are more compact compared to DNA thanks to backbone interactions mediated by ribose zippers.^37^ To date, only one study has demonstrated the potential of RNA as a building block for membrane pores. Li et al.^24^ reported the assembly of RNA nanotubes from multiple RNA strands.

Despite their advantages, small nucleic acid nanopores also have some limitations. These include structural instability in lipid bilayers, ion leakage around the pore’s perimeter, and low membrane insertion efficiency due to the often unfavorable balance of number of anchors and pore perimeter on scaffold-free designs.^21,33,38^ Ongoing research continues to address these challenges through strategic chemical modifications and improved design principles.

### 2.2 Medium-Diameter DNA Nanopores (4–10 nm)

While the compact architecture of small DNA nanopores provides size exclusion and molecular selectivity, larger designs have been developed to accommodate broader functional demands. Medium-sized DNA nanopores, typically 4–10 nm in diameter, offer enhanced ionic conductance and expanded capabilities for translocation of larger cargo molecules. They bridge the gap between small selective channels and large membrane-spanning assemblies, enabling diverse applications in biosensing, molecular filtration, and controlled transport.

A series of notable developments highlight the capabilities of medium-sized DNA nanopores. In 2016, Göpfrich et al.^17^ achieved a breakthrough with their funnel-shaped DNA origami porin featuring a 6 nm nominal cross-section, the largest synthetic pore in a lipid membrane at that time. The structure incorporated 19 cholesterol tags for membrane anchoring and exhibited exceptionally high conductance levels of 30 nS in 1 M KCl, surpassing small DNA nanopores by an order of magnitude. Concurrent with this development, Krishnan et al.^11^ introduced a T-pore architecture, comprising a double-layered rectangular plate connected to a hollow transmembrane stem with a 4.2 nm diameter (Figure 1f). This particular design demonstrated high membrane insertion frequency and maintained a consistent conductance of ∼ 3.1 nS without spontaneous gating hinting toward good structural stability. Importantly, the T-pore’s enlarged diameter enabled the translocation of both ssDNA and double-stranded DNA (dsDNA) molecules, transcending the limitations of smaller nanopores that typically accommodate only ions and ssDNA if an external electric field is applied to drive ssDNA translocation. Further advancing the field, Thomsen et al.^12^ developed a sophisticated 9 nm-diameter, double-layered hexagonal DNA nanopore. These designs incorporated multiple functionalization sites within the channel for biomolecular interactions and featured innovative programmable lipidated flaps. These flaps remained in a locked state until activated through toehold-mediated strand displacement, enabling sequence-specific sensing capabilities. The incorporation of movable design elements positioned the nanopore as a powerful tool for precise nucleic acid discrimination and biomolecular diagnostics.

The emergence of medium-sized DNA nanopores has opened new possibilities across various applications. Their enhanced capacity to accommodate larger molecules while maintaining high conductance and structural stability makes them valuable candidates for single-molecule sensing,^11^ particularly in studying molecular interactions and complexes. Furthermore, their spontaneous insertion into lipid bilayers, as visualized in many studies above through giant unilamellar vesicles (GUVs) experiments,^11,12^ suggests promising applications in synthetic biology and drug delivery systems. However, several challenges persist in this field. The size-selective nature of molecular transport in medium-sized nanopores can lead to pore blockage or clogging by larger biomolecules, impeding effective single-molecule analysis. In complex biological environments, the presence of these large molecules may further interfere with the detection of smaller analytes, compromising overall sensing efficiency. Additionally, the translation of these technologies to clinical and industrial applications demands higher throughput, necessitating continued optimization of pore design to enhance efficiency and improve integration with biological systems.

### 2.3 Large-Diameter DNA Nanopores (>10 nm)

The emergence of large DNA nanopores represents a significant leap forward in synthetic channel design, addressing challenges inherent to both natural protein channels and smaller synthetic pores. While nanopores with widths of several nanometers enable the electrophoretic sensing of similarly sized DNA strands, small organic molecules and proteins under an applied voltage, their confined geometries restrict the passage of larger biomolecules. Few protein pores used for sensing applications have diameters over 10 nm because large protein channels are extremely rare and often difficult to reconstitute functionally.^39^ Consequently, synthetic pores remain an effective option for the controlled transport of larger molecular cargo. Importantly, wide nanopores are also advantageous in systems relying on passive diffusion without an external electric field, as access resistance renders passive transport highly inefficient when the cargo size approaches the pore diameter.^40^ These limitations have motivated the development of programmable synthetic nanopores specifically tailored for the efficient translocation of large, biologically relevant molecules. Such wide channels hold transformative potential for single-molecule analysis of large enzymes, immunoglobulins, protein complexes, and even viruses. Beyond simple cylindrical geometries inspired by biology, synthetic DNA nanopores can also be engineered in diverse architectures, further expanding their functional versatility. For example, Dey et al.^18^ designed a sophisticated reversibly gated protein-transporting membrane channel with a square-shaped lumen measuring an impressive 20.4 nm × 20.4 nm (Figure 1g). This channel featured a nanomechanical lid that could be selectively closed and reopened via a lock-and-key mechanism, enabling precisely timed, stimulus-controlled transport of folded and functional proteins across bilayer membranes. Another large-diameter DNA nanopore design was introduced by Xing et al.,^31^ who arranged bundled DNA duplex subunits to construct quadrilateral nanopores with customizable side lengths of 10 and 20 nm. This modular design strategy provided fine control over pore shape and size, further facilitating the creation of multiple tunable geometries, including triangles, pentagons, and hexagons, with lumen areas ranging from 43 nm^2^ to 400 nm^2^ (Figure 1h). Pore modifications enabled excellent biosensing capabilities, such as the detection of IgG and SARS-CoV-2 antibodies, showcasing their potential for clinical and high-throughput diagnostics. Liu et al.^32^ developed a triangular DNA nanopore that could switch between an expanded (45 nm side length) state and a contracted (30 nm side length) state by adjusting the relative positions and lengths of structural components. This tunable design enables configuration-dependent size selective transport, providing a significant advantage over traditional static nanopores for responsive biosensing platforms.

These large DNA nanopores represent a transformative advance over traditional protein nanopores which typically have diameters of only a few nanometers. The expanded dimensions of DNA nanopores enable the transport and sensing of much larger biomolecules like folded proteins and antibodies in their native form or even as part of larger complexes. The DNA assembly enables precise geometric control and the ability to incorporate functional elements like molecular recognition sites and stimuli-responsive components and unlocks new possibilities in biosensing, controlled drug delivery, and synthetic biology.

Notably, these DNA nanopores have demonstrated compatibility with both research-grade recording setups and portable devices like the MinION sequencer,^31^ highlighting their practical utility. The combination of large size, tunable geometry, and molecular-scale control establishes DNA nanopores as a powerful tool for studying and manipulating biological systems.

## 3 Interactions Between Nucleic Acids and Membranes

### 3.1 Energetics of Membrane Insertion

Insertion of a transmembrane pore into a lipid membrane requires overcoming an energetic barrier. For a cylindrically symmetric pore, the free energy of pore formation—and thus the insertion barrier for a nanopore—is commonly described by two contributions. The first term represents the energetic cost of creating the pore edge and is given by the product of the pore perimeter 2π*r* and the line tension γ, which has been shown to depend on both the pore radius and the lateral tension, γ­(*r*, σ_0_).^41^ In the classical theory of pore formation,^42^ the line tension also accounts for the unfavorable exposure of the hydrophobic membrane core to the aqueous phase, as well as the elastic deformation of the membrane at the pore rim that enables formation of a toroidal edge and thereby reduces such exposure. The second term accounts for the energetic gain associated with tension release and is given by the product of the pore area, π*r* ^2^, and the lateral tension, σ_0_.

1 $$E\left(r\right)=2\pi r\gamma \left(r,\sigma_{0}\right)-\pi r^{2}\sigma_{0}$$

The magnitude of the bending penalty and, more generally, the feasibility of toroidal pore formation depends strongly on the membrane material properties and on the geometry of the inserted structure. In particular, lipid fluidity and lipid shape critically influence how efficiently the bilayer can reorganize around a nucleic acid pore. Membrane fluidity describes both the degree of disorder and the rate of lateral diffusion of membrane constituents^43^ and is largely governed by lipid structure, especially the saturation of the fatty-acid tails. Unsaturated tails disrupt van der Waals interactions between lipids, increasing disorder and enhancing membrane flexibility.^38^ In more fluid membranes, lipids can rearrange more readily to accommodate nanopores, which can reduce local stress and promote stable insertion. Conversely, in more ordered phases (e.g., raft-like domains), insertion may drive local disruptions, including domain segregation or nanopore clustering.^44^

Lipid reorganization during DNA nanopore insertion is also strongly size-dependent. Small pores (1–2 nm in diameter) can insert via transient bilayer defects, with lipids reorganizing locally around the hydrophilic nucleic acid structure. In contrast, larger pores (10–30 nm) require substantial lipid displacement, which can lead to pronounced remodeling such as bilayer thinning, lipid extraction, or pore-induced phase separation.^45^ For pores exceeding ∼30 nm, the energetic cost rises steeply, often necessitating assisted insertion methods such as droplet-interface crossing encapsulation (cDICE), in which the origami pore is first inserted into a lipid monolayer, followed by bilayer formation.^45^

Unlike biological protein pores, which typically overcome the energetic barrier to insertion by embedding hydrophobic regions into the hydrophobic core of the membrane and utilizing specific folding mechanisms,^46^ nucleic acid nanopores require alternative strategies. Therefore, nucleic acid pores rely on membrane anchors to achieve stable insertion into lipid bilayers, preserve structural integrity, and enable controlled molecular transport.^47^ A membrane anchor must provide an energetically favorable interaction with the membrane, either with the hydrophobic core, the hydrophilic head groups, or with other membrane components or modifications (e.g., proteins, peptides, biotin, sugars). The total energy provided by all anchors must exceed the energetic barrier for stable pore formation. In addition, the anchors must be positioned in a symmetric configuration to ensure torque balance, which is essential for long-term stability. Moreover, the linkers that connect the anchors to the nucleic acid pore itself cannot be too long and have to be positioned such that they can only reach the membrane when the pore is correctly inserted. In other words, there has to be a net energy gain for insertion of the pore compared to it lying flat on the membrane.

### 3.2 Membrane Anchoring Strategies

Various chemical modifications and structural adaptations have been developed to overcome the insertion energy barrier of nucleic acid transmembrane pores and to achieve stable membrane anchoring.^33^ Beyond these established strategies, which require chemical modifications of the nucleic acid, we also review interactions between membranes and unmodified DNA or RNA and discuss their potential as natural anchoring mechanisms for future applications.

#### 3.2.1 Electrostatic Membrane Interactions

Interactions between unmodified nucleic acids and membranes are predominantly driven by electrostatic forces. While binding between a net positively charged membrane and the polyanionic nucleic acid is straightforward, interactions with zwitterionic or negatively charged membranes are also possible. In such cases, binding is typically mediated by divalent cations, which bridge the negatively charged phosphate groups of DNA or RNA and those of membrane lipids—often phospholipids (Figure 2 (i)). In contrast, monovalent cations can shield negatively charged groups, thereby preventing charge bridging. Consequently, the ratio between divalent and monovalent ions is a critical parameter for nucleic acid–membrane interactions.^48^

**Figure 2:**
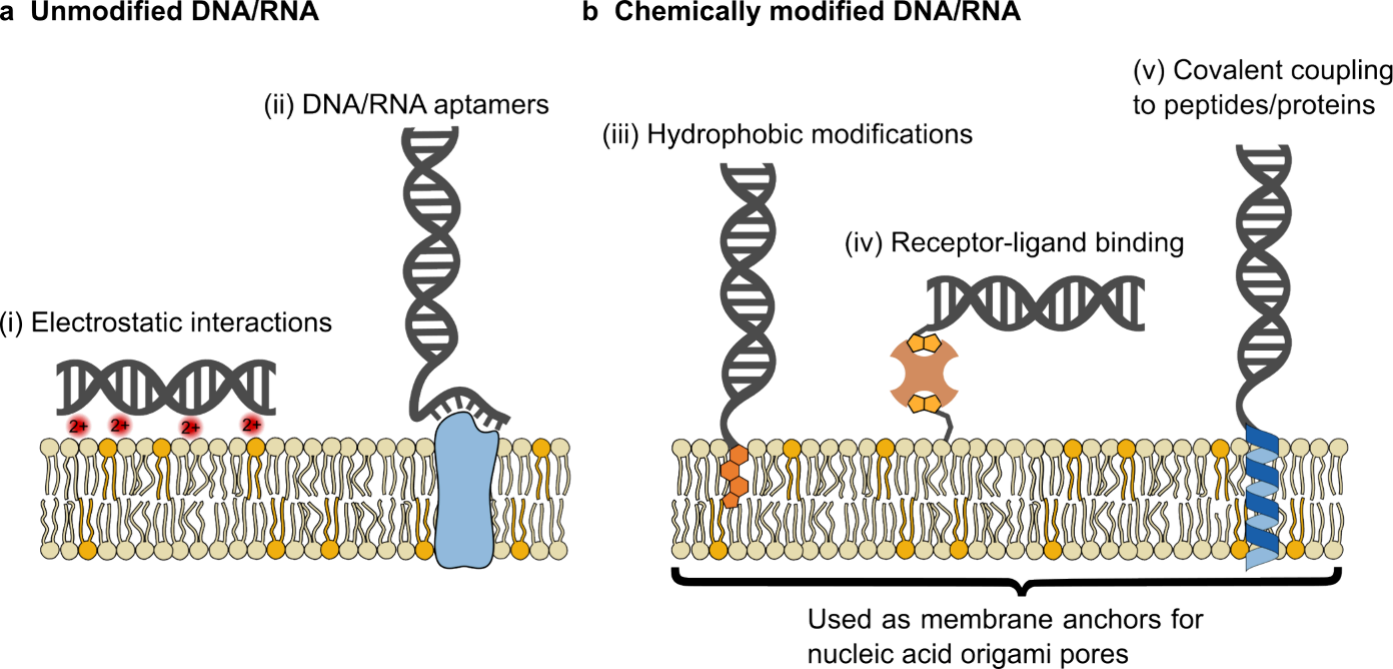
Interactions between nucleic acids and lipid membranes. (a) Unmodified DNA or RNA interact with membranes mainly via (i) electrostatic interactions, directly with positively charged lipids, or with zwitterionic/negatively charged lipids in the presence of divalent cations, while monovalent cations tend to inhibit binding,^48–50^ (note that for RNA, hydrogen bonding may also contribute) and (ii) sequence specific interactions of aptamers binding to lipid headgroups or membrane components such as proteins or biotin.^54,55^ Both interaction types of unmodified nucleic acids have been used to bind nucleic acid structures to membranes, but not yet as anchors for nucleic acid pores. (b) Chemical modification of nucleic acids further expands their modes of membrane interaction. By far the most widely used approach for nucleic acid pore insertion into lipid membranes is (iii) the introduction of hydrophobic modifications, for example cholesterol, tocopherol, poly­(propylene oxide), porphyrins, single-chain fatty acids, or steroids, which insert into the bilayer core and thereby mediate robust membrane attachment.^38^ Another strategy exploits (iv) receptor–ligand interactions, such as the strong and specific biotin–streptavidin binding, in which streptavidin bridges biotinylated DNA and biotinylated lipids to anchor DNA origami pores.^11^ DNA or RNA can also be (v) covalently coupled to membrane active proteins or peptides that insert into lipid bilayers, thereby anchoring peptide/protein–DNA hybrid pores to membranes.^56–59^

The strength of nucleic acid–membrane interactions also depends strongly on membrane order, i.e., the gel–fluid state. Higher lipid order generally increases the likelihood of binding, and the following trend has been reported: liquid disordered (*L*_*d*_) < liquid ordered (*L*_*o*_) < ripple state (*P*_β′_) < gel state (*L*_β_).^48–51^ This poses a challenge for exploiting electrostatic interactions as membrane anchors because the energetic cost of insertion follows the opposite trend, as membrane remodeling and deformation are more difficult in rigid membranes.

However, the strength of electrostatic interactions also depends on the length and stiffness of the nucleic acid. For DNA, the following general order has been reported: ssDNA < dsDNA < long ssDNA < long dsDNA < DNA origami.^48^ Although no equivalent systematic study has been conducted for RNA, it is reasonable to assume that RNA–membrane interactions would follow a similar trend. A crucial difference, however, is the presence of an additional 2’–OH group in RNA. This group could, potentially, allow for extra hydrogen bonding with lipid head groups.^52,53^ The fact that RNA membrane interactions could be favored over those of DNA makes RNA an interesting target for future nanopore designs.

To date, no nucleic acid pores relying solely on purely electrostatic membrane anchors have been reported, and it remains unclear whether such an approach would be feasible. One conceptual design might involve a square pore surrounded by a flat origami sheet, similar to designs that used other forms of anchors;^11^ however, such a structure would require modifications to prevent preferential side binding on the flat side without channel insertion. A simple modification would be to extend the channel so that it protrudes symmetrically from both sides of the membrane. Nevertheless, whether the energy provided purely by electrostatics would be sufficient for stable insertion of the pore into the membrane—even under optimal ionic conditions—remains questionable and would most likely require the use of conical lipids to aid toroidal pore formation. Electrostatics might instead be best considered as one factor contributing to better membrane recruitment and thus higher insertion efficiencies in combination with other membrane anchors.

#### 3.2.2 Sequence-Specific Membrane Interactions

Another type of membrane interaction without chemical modification of the nucleic acid is the sequence-specific binding of DNA or RNA to membranes or membrane components (e.g., proteins, peptides, or sugars) (Figure 2 (ii)). Such aptamers are generally obtained through Systematic Evolution of Ligands by EXponential enrichment (SELEX),^60^ a directed evolution experiment whereby a library of up to 10^16^ different sequences is exposed to a target (here a membrane). The successfully binding fraction is extracted from the mixture and subjected to sequencing or another round of selection. A notable series of studies by Michael Yarus and colleagues (1999–2006) identified RNA species capable of binding membranes.^49,54,61–64^ Using size exclusion chromatography, they found that a subset of RNAs coeluted with small liposomes, whereas random, nonbinding RNAs appeared in later fractions. The minimal membrane-binding unit comprised two distinct RNA species which formed multimeric complexes.^54^ Certain sequence regions were proposed to mediate RNA–RNA interactions, while others were suspected to participate in membrane binding; however, the exact mechanism remained unclear. Binding was shown to be enhanced by divalent cations (Ca^2+^ > Mg^2+^) and reduced by monovalent cations (Na^+^),^54^ indicating that the sequence specific binding also has a significant electrostatic component. Interestingly, later studies reported that a specific RNA sequence, termed RNA 10, alone could bind to negatively charged lipids even in the absence of ions, suggesting a more complex, aptamer-like interaction mechanism.^65^ In another study, the RNA sequence was further modified with an additional aptamer for tryptophan, which increased the influx kinetics of tryptophan across a membrane.^64^

Another study identified long noncoding RNAs that associate with lipid fractions isolated from patient-derived cancer tissue.^66^ Of nine candidates, seven exhibited specific enrichment for distinct phospholipid species, most notably phosphatidylcholine (PC) and phosphatidylinositol-3,4,5-trisphosphate (PIP_3_). The most prominent lipid-binding RNA, termed LINK-A, is 1,540 nt in length. Distinct 60 nt subsequences mediating binding to PC and PIP_3_ were mapped. Full-length LINK-A bound PIP_3_ with a dissociation constant (*K*_*d*_) of 112 ± 37 nM, comparable to or stronger than many protein–PIP_3_ interactions, whereas a minimal 18-nt stem-loop motif was sufficient for PIP_3_ binding but displayed lower affinity (*K*_*d*_ ≈ 2 μ M).

Recently, it was shown that interactions between nucleic acids and gel-phase lipid membranes, long considered sequence-unspecific, are in fact strongly dependent on nucleotide composition. Czerniak and Saenz^51^ demonstrated that short guanine oligomers bind much more strongly to gel-phase membranes than oligomers of any other nucleotides. Conversely, depletion of guanine from a mixed 40-nt oligomer led to the strongest reduction in binding, by approximately 1 order of magnitude. DNA-based aptamers for lipids have not been reported, likely because of the more limited structural diversity of DNA.

Importantly, nature itself provides a precedent for RNA functioning as an integral membrane component. Recent structural and functional studies of OLE RNA (ornate, large, extremophilic RNA) suggest that this highly conserved bacterial noncoding RNA forms a membrane-associated ribonucleoprotein complex and may span the lipid bilayer, making it one of the first known examples of a naturally occurring integral membrane RNA.^23^ Interaction between OLE RNA and OapA (OLE-associated protein A) is required for OLE RNA to localize to the host-cell membrane. Furthermore, the authors speculate that regions of OLE RNA that reside in or near the membrane environment could interact in a sequence-specific manner with membrane proteins (in addition to OapA) or with the lipid bilayer itself. These findings support the notion that RNA can, in principle, adopt architectures compatible with stable membrane insertion and transmembrane function, providing strong biological support for the feasibility of RNA-based membrane pores.

Alternatively to direct interactions with lipids, aptamers that interact with lipid headgroup modifications, such as biotin aptamers,^55,67^ have been used to anchor RNA nanostructures on lipid membranes.^68,69^ Such strategies could potentially be used in the future to overcome the energy barrier for pore insertion.

To the best of our knowledge, sequence-specific membrane interactions have not been employed as membrane anchors for synthetic nucleic acid pores. However, the use of nucleic acid structures appears promising. RNA in particular offers strong opportunities in this regard, and it seems likely that future nucleic acid pores could incorporate selected aptamers—either directly targeting lipid headgroups, membrane proteins or other membrane components. This could be implemented using DNA aptamers in a DNA origami pore, or more plausibly, RNA aptamers in a DNA–RNA hybrid pore or a fully RNA origami pore.

#### 3.2.3 Hydrophobic Interactions

Currently, most published DNA and RNA nanopores were inserted into lipid membranes by using chemical modifications covalently linked to the DNA/RNA. Among these modifications, the most common membrane anchors rely on hydrophobic interactions (Figure 2 (iii)).

Various hydrophobic moieties, including cholesterol, tocopherol, poly­(propylene oxide), porphyrin, single chain fatty acids, and steroids, have been conjugated with DNA strands to facilitate membrane attachment.^38,70^ Among these, cholesterol is the most widely used membrane anchor for nucleic acid pores due to its high affinity for lipid bilayers and its commercial availability as a 5′- or 3′-modification on oligonucleotides.

When cholesterol-modified nucleic acid nanostructures encounter lipid membranes, the hydrophobic cholesterol units spontaneously insert into the bilayer, ensuring strong and stable attachment. Note that even here, divalent cations such as Mg^2+^ are often required to be present in the binding buffer,^11,16^ indicating that electrostatic interactions might still assist with the membrane interactions, particularly during the initial membrane-approach and attachment stages. The strength of cholesterol-mediated membrane anchoring can be enhanced through multiple cholesterol moieties, which effectively strengthen lipid interactions and improve membrane insertion efficiency. However, a balance must be maintained, as excessive cholesterol modifications can lead to aggregation and potentially hinder membrane integration. This challenge may be addressed by optimizing spacer lengths and strategic positioning of cholesterol groups, which further enhance the stability of membrane attachment.^71–73^ Additionally, it has been shown that aggregation can be minimized by single-stranded overhangs extended adjacent to the cholesterol, as MD simulations indicate that these overhangs wrap around the cholesterol, effectively shielding its hydrophobicity until the membrane is reached.^73^ Beyond membrane anchoring, cholesterol-functionalized DNA nanostructures offer an additional advantage in cellular uptake through endocytosis-mediated internalization,^8^ making them particularly valuable for drug delivery and intracellular biosensing applications.

In addition to cholesterol, tocopherol has emerged as an effective alternative for integrating DNA nanopores into lipid membranes leveraging hydrophobic interactions. It naturally integrates into liquid-disordered regions,^9^ enabling selective localization in dynamic cellular environments such as endocytic pathways. This anchoring strategy preserves DNA functionality, allowing for hybridization and molecular recognition while maintaining membrane integrity with minimal perturbation. Furthermore, Tokunaga et al.^74^ demonstrated that tocopherol, as a nontoxic lipophilic molecule, naturally adheres to cellular membranes, allowing DNA conjugated to α-tocopherol at the terminal phosphate moiety to spontaneously insert into cell membranes without the need for chemical preactivation. This property significantly simplifies the process of anchoring DNA-based sensors or nanostructures to biological membranes while preserving their functional integrity. Kurz et al.^75^ further found that α-tocopherol units could not only facilitate membrane integration but also enhance the stability of double-helix formation within distinct membrane domains. Their findings highlight the preferential localization of tocopherol-anchored DNA in liquid-disordered regions, providing a stable and adaptable platform for membrane-bound DNA nanostructures.

Another significant example of hydrophobic anchoring in DNA nanopores is porphyrin and its derivative, tetraphenyl porphyrin (TPP). Despite its size, shape, and hydrophobicity being similar to cholesterol, porphyrin possesses a high van der Waals surface area, which can be further increased with additional aromatic substituents. This enhanced hydrophobicity enables DNA structures to insert into lipid bilayers with minimal chemical modification.^13^ It is worth noting that porphyrin serves not only as a functional anchor for ligand docking sites that enable potential coordination interactions, but also as a versatile electron and energy transfer component, making it a powerful tool for fluorescence visualization and experimental tracking.^13,15^ Börjesson et al.^76^ further demonstrated that porphyrin is particularly well-suited as a photophysical and redox-active lipid anchor, offering a significant advantage over the inert cholesterol anchor. The unique combination of hydrophobic anchoring and optoelectronic capabilities makes porphyrin a highly promising component for membrane-integrated nanodevices, biosensing platforms, and potential energy-harvesting applications.

A particularly notable advancement in hydrophobic anchoring was introduced by Burns et al.,^13,14^ who demonstrated that direct alkylation of DNA nanostructures with short ethyl groups can provide a continuous hydrophobic surface around the nanopore body. Unlike anchor-based approaches (e.g., cholesterol or porphyrin) that primarily localize hydrophobicity at specific termini or junctions, this strategy introduces a hydrophobic belt around the entire DNA pore periphery. This modification enables stable and detergent-free insertion of the nanopore into lipid membranes without the need for bulky external anchors. Furthermore, MD simulations revealed that lipid headgroups reorganize around these alkyl-modified surfaces, tightly wrapping and intercalating between the helices to prevent lateral ion leakage and enhance membrane compatibility. A hydrophobic belt is particularly advantageous in mimicking natural transmembrane proteins, where hydrophobic exterior surfaces are essential for long-term membrane integration. The ethyl modification thus represents a minimalistic yet effective design principle, extending the capabilities of DNA nanopores for functional integration into biological and synthetic membranes alike.

However, the predominant approach for DNA nanopores remains the addition of hydrophobic molecules. The focus on DNA with anchors is mainly driven by the easy (commercial) access of modified DNA molecules. Making the outside of the DNA nanopore hydrophobic through modifications of the backbone might reduce the ion leakage along the highly charged backbone. It would be interesting to explore other backbone chemistries like peptide nucleic acids (PNA). DNA assembly and handling may be profoundly changed in that case and require more basic characterization of the assembly pathways.

#### 3.2.4 Receptor–Ligand Interactions

It is important to note that hydrophobic interactions are not the only way to insert DNA nanopores into lipid membranes. If the interaction between the headgroups and the DNA is large enough, it is possible to overcome the energy barrier for insertion of the hydrophilic stem. This can be achieved by receptor–ligand interactions on the membrane surface, as has been demonstrated with a biotin-modified DNA pore (Figure 2 (iv)).^11^ In this system, biotinylated DNA strands are incorporated into a DNA origami nanopore and bind to biotinylated lipids in the membrane in the presence of the ligand streptavidin. Since streptavidin is a tetrameric protein with four high-affinity biotin-binding sites, it acts as a molecular linker, inserting the DNA nanopores into the lipid bilayer.

One of the primary advantages of biotin-mediated anchoring over hydrophobic interactions is its exceptional stability. The biotin–streptavidin bond is among the strongest known noncovalent interactions, with a dissociation constant in the femtomolar range (∼4 × 10^–14^ M),^77^ ensuring that DNA nanopores remain stably attached even in dynamic biological environments. In contrast, hydrophobically modified DNA anchoring, which typically presents a dissociation constant in the micromolar to nanomolar range,^78^ provides effective membrane insertion but relies on inherently reversible interactions. These interactions are sensitive to lipid phase separation, leading to potential detachment and making it less suitable for applications requiring prolonged stability and persistent functionalization.^74^

Another notable advantage of biotin-based anchoring is that the structures are less prone to aggregation, a common challenge associated with hydrophobic anchors. Biotinylated lipids can be readily incorporated into synthetic vesicles, supported lipid bilayers, and even live cell membranes,^11^ making it suitable for a wide range of both *in vitro* and *in vivo* applications.

Despite these advantages, unlike hydrophobic anchors such as cholesterol or tocopherol, which enable spontaneous membrane insertion upon contact with a lipid bilayer, biotin anchoring requires a multistep process. This involves prefunctionalization of the membrane with biotinylated lipids and subsequent streptavidin-mediated bridging to biotinylated DNA. As a result, biotin anchoring is less direct and experimentally more involved than hydrophobic anchoring strategies. The requirement for prefunctionalized membranes also restricts its applicability in natural biological systems where biotinylation may not be feasible or desirable. Additionally, while the near-irreversible nature of biotin–streptavidin binding is beneficial for maintaining stable nanopore attachment, it can be a disadvantage in applications that require transient or tunable membrane interactions. In contrast, both cholesterol and tocopherol provide reversible binding, allowing for controlled dissociation and reattachment by modifying lipid composition or environmental conditions.

Another challenge of biotin anchoring is steric hindrance, as streptavidin is a relatively large protein (∼5 nm in diameter^79^). In high-density DNA nanopore arrays, closely packed biotinylated structures may experience crowding effects, reducing effective binding efficiency and potentially obstructing functional nanopore sites.

Apart from the biotin–streptavidin system, we are aware of only one other receptor–ligand interaction that has been employed to anchor nucleic acid pores to membranes. In a recent study, the antibody trastuzumab was conjugated to a DNA pore (“DNA origami needle”) to enable specific binding and insertion into HER2 receptor–positive cells.^80^ However, in this case, the antibody functioned only as a secondary membrane anchor in combination with cholesterol. Importantly, the authors showed that efficient and specific binding, as well as pore insertion, required the presence of both the antibody and cholesterol. Although antibodies remain relatively unexplored as membrane anchors for nucleic acid pores, they represent a highly promising direction—particularly for applications where cell-type–specific pore insertion is desired. Furthermore, coupling DNA to antibodies is a well-established strategy in other contexts, such as DNA-PAINT,^81^ where it has also been applied to target membrane proteins in both *in vitro* systems and live cells.^82^

At the time of writing, other receptor–ligand interactions have not yet been systematically explored for membrane insertion, but in principle this strategy could be extended to a variety of systems. The choice of anchoring mechanism will depend on the intended application and requires balancing stability, reversibility, specificity, and ease of integration.

#### 3.2.5 Nucleic Acids Coupled to Proteins or Peptides

Lastly, nucleic acids can also be covalently coupled to membrane-active proteins or peptides, yielding peptide/protein–DNA hybrid nanopores (Figure 2 (v)). Henning-Knechtel et al.^56^ templated α-hemolysin monomers on circular DNA to enforce non-native oligomeric stoichiometries (e.g., 12-, 20-, and 26-mers), yielding stable insertions and pore size-dependent conductance levels. In a related strategy, a DNA origami ring was used to position up to 48 copies of the cholesterol-dependent toxin pneumolysin (PLY), generating uniformly sized channels with an average inner diameter of ∼22 nm and enabling size-dependent exchange of macromolecules. Decorating the DNA ring with intrinsically disordered nucleoporins further increased size selectivity.^57^ Moving from proteins to peptides, DNA-programmed assembly of the pore-forming peptide ceratotoxin A (CtxA) on templates with 4, 8, or 12 binding sites produced pores spanning ∼0.5 to 4 nm in diameter and enabled modular functionalization such as DNA-cholesterol anchors to increase membrane affinity and modifications that prolong open-state lifetimes. DNA templating of CtxA also enhanced cytotoxicity, as shown by a comparable cancer-cell killing rate at ∼20-fold lower total peptide concentration compared to the nontemplated peptide.^58^ Finally, DNA scaffolds have been used to stabilize peptide nanopores derived from the octameric polysaccharide transporter Wza: scaffolded assemblies formed uniform, conducting pores, whereas cleavage of the DNA-peptide linkers caused rapid loss of the pores from the membrane, underscoring the scaffold’s stabilizing role.^59^

However, it is important to emphasize that in these peptide/protein–DNA hybrid nanopores, the peptide or protein constitutes the membrane pore, whereas the nucleic-acid component primarily acts as a geometrically defined scaffold that controls the stoichiometry and pore diameter and provides handles for further functionalization. Since the focus of this review is on nucleic-acid pores as the membrane-spanning transport pathway, these hybrids are not discussed further here. Nevertheless, membrane-active peptides or proteins could, in the future, be repurposed as modular anchors for nucleic-acid nanopores, enabling DNA or RNA origami to form the actual membrane-spanning channel while peptide/protein moieties provide membrane affinity and insertion assistance. A more detailed overview of DNA-functionalized protein and peptide nanopores is provided by Xing et al.^1^

### 3.3 Factors Influencing Insertion Efficiency

The efficiency of DNA nanopore anchoring to lipid membranes is governed by multiple interdependent factors, including membrane composition, fluidity and curvature, pore diameter, buffer conditions, anchor density and positioning, aggregation effects and spacer length. Each of these parameters plays a crucial role in determining the success of membrane attachment, the insertion rate and the functional stability of the nanopore. To understand how to design a DNA nanopore which inserts as efficiently as possible into lipid membranes, let us consider the steps of the insertion process:

#### 3.3.1 Before Attachment and Insertion

The DNA/RNA pore has to freely diffuse in aqueous solution until it encounters the lipid membrane. Here it is beneficial if the pore does not aggregate in solution. While a higher number of hydrophobic tags helps to get to a net energy gain during the insertion, it is not always beneficial for the insertion rate since it also increases the propensity of the pores to aggregate. The number of anchors should thus be tuned to match the pore size. The required number of cholesterol tags for a given pore diameter has been determined by Göpfrich et al.^17^ To reduce aggregation, hydrophobic tags can additionally be shielded, for instance by ssDNA overhangs.^73^ Alternatively, receptor–ligand strategies which do not involve hydrophobic moieties, such as biotin–streptavidin linkage^11^ can be used.

#### 3.3.2 Attachment

Attachment of the DNA pores always has an electrostatic contribution, hence buffer conditions need to be tuned accordingly. Divalent ions are typically needed for efficient attachment. On the contrary, monovalent ions reduce electrostatic interactions and yet they are often required for single channel ionic current recordings. For many designs, attachment likely means that the pores first lie flat on the membrane. Furthermore, attachment of nucleic acids to liquid ordered membranes is favored compared to liquid disordered membranes, however, this likely has no benefit for insertion, as lipid reorganization is more challenging to achieve when membranes are in the ordered state.

#### 3.3.3 Insertion

Insertion is energetically possible if the pores are designed such that all membrane anchors can only insert into the membrane when the stem pierces the membrane. Here, it is particularly important to consider linker length (e.g., cholesterol is often attached to DNA via longer linkers). When the pores lie flat on the membrane, not all anchors can reach the membrane—otherwise there is no net energy gain for stem insertion. Too short spacers, however, may restrict the flexibility of the nanopore, creating steric hindrance that reduces insertion efficiency. Thermal fluctuations induce repositioning of the pore, and once enough anchors reach the membrane, the stem starts to be forced into the membrane. At this point, the lipids reorganize such that the hydrophilic head groups face the pore. As soon as the pore is inserted in the perpendicular membrane-spanning orientation, all anchors are in contact with the membrane and the lipids have fully reorganized to form a toroidal pore which transports lipids from one side to the other. Pore geometry can be exploited to enhance insertion: Geometries like funnel shapes initially only require little membrane remodelling. It remains unclear, however, how other design choices, like pore rigidity, helix interconnectivity and fraying of the helices at the pore ends^83^ influence insertion.

#### 3.3.4 After Insertion

For most applications, in particular for sensing, membrane insertion should be stable over prolonged periods of time. Hence, strong interactions are favored and can be achieved e.g. with biotin–streptavidin anchoring. On the other hand, if tunability and stimuli-response are desired, stimuli-responsive anchoring strategies have to be considered (e.g., strand displacement, environmentally sensitive cholesterol anchoring). Optimizing DNA nanopore anchoring therefore requires a delicate balance between stability, specificity, reversibility, and membrane compatibility.

### 3.4 Transport Pathways Mediated by Nucleic Acid Nanopores

Nucleic acid origami nanopores can facilitate multiple modes of transport for ions, macromolecules, and lipids across—and along—the lipid membrane. A key determinant is the chemical nature of the nanopores, in particular the hydrophilicity of the sugar–phosphate backbone of the nucleic acids, which is polar and strongly hydrated in aqueous environments. As a result, the hydrophilic pore lumen enables the passage of ions and hydrated macromolecules across the membrane.^84^ Size selectivity for macromolecular transport can be achieved by tuning the lumen diameter.^45^

In contrast to protein nanopores, which typically insert into lipid membranes via direct hydrophobic interactions of membrane-facing hydrophobic amino acids arranged to form a well-defined transmembrane domain^85^ (Figure 3a), nucleic acids do not possess the same chemical versatility as amino acids. While the hydrophilic nature of nucleic acids is advantageous for maintaining a hydrated pore lumen, it also forces the lipid bilayer to adopt a toroidal geometry by reorienting hydrophilic lipid headgroups toward the pore. Beyond its energetic implications for pore insertion and stability discussed above (Section 3.1), a thin hydration layer persists between the membrane and the nucleic acid helices, which is sufficient to support ion passage (Figure 3b). This was confirmed both by MD simulations and experimentally^30^ in a study by Göpfrich et al. in which they inserted a single DNA duplex into a lipid membrane and observed discrete steps in ion conductivity across the membrane. As this minimalistic pore design does not feature a pore lumen, ion passage can only take place on the interface between the membrane and nucleic acid.^30^

**Figure 3:**
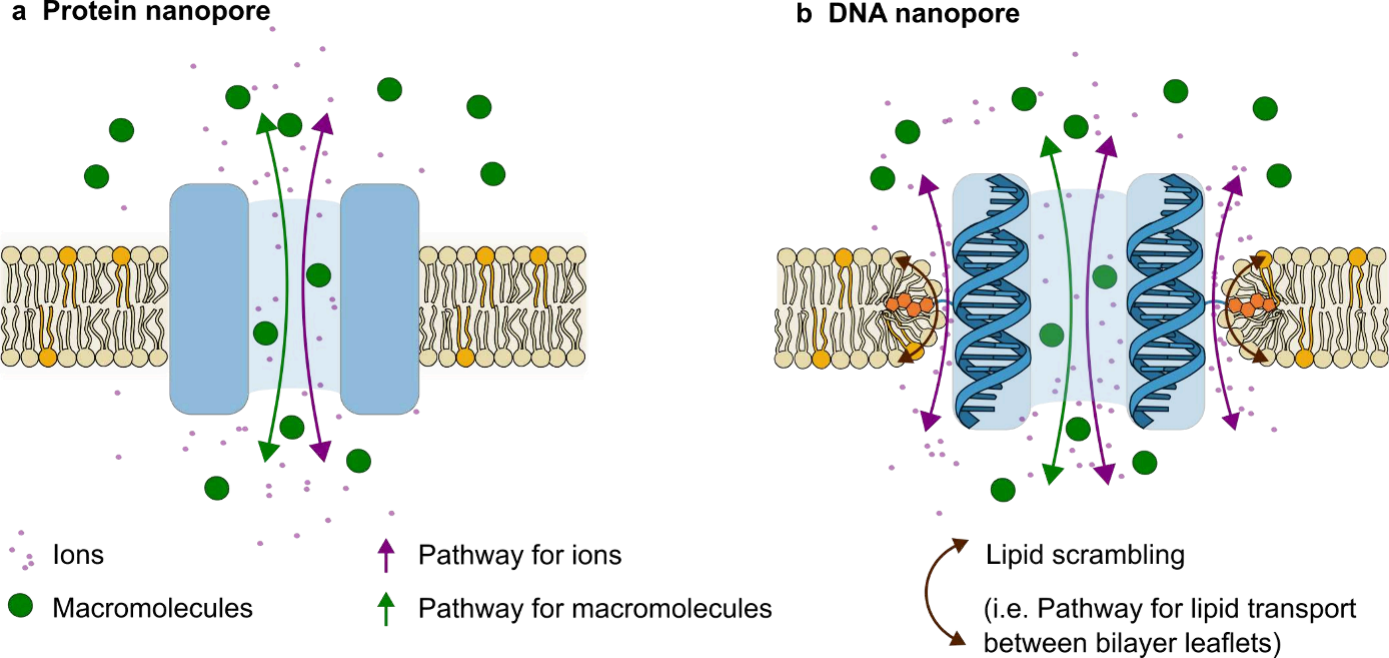
Transport pathways across lipid membranes through protein and DNA nanopores. (a) Protein pores allow ions and macromolecules to diffuse through the pore lumen and are inserted into the lipid bilayer via hydrophobic interactions between amino acids and the membrane core. (b) DNA nanopores also permit diffusion of macromolecules and ions. When the negatively charged DNA is inserted into the membrane, a toroidal lipidic pore is formed, allowing ions to leak through the interface between DNA helices and lipid headgroups,^30^ and enabling lipid diffusion between bilayer leaflets, resulting in scramblase activity.^86,87^

If such ion leakage is undesired, direct alkylation of DNA nanostructures with short ethyl groups (as discussed above in Section 3.2.3) can be used to prevent toroidal pore formation and thereby suppress ion leakage across the nucleic acid–membrane interface.^13,14^

The topological change associated with a toroidal pore, which connects the two membrane leaflets, also enables an additional pathway: lipid transport by lateral diffusion. In cells, bidirectional translocation of phospholipids across the lipid bilayer is facilitated by membrane protein scramblases. A nucleic acid pore that induces a toroidal membrane shape should therefore exhibit scramblase-like activity. Indeed, experiments and simulations have confirmed scramblase-like activity of DNA nanopores.^86,87^

Membrane curvature critically influences both the efficiency of nanopore insertion and the subsequent stability of membrane-bound pores.^44,88^ Highly curved membranes, such as small unilamellar vesicles (SUVs)^12^ or liposomal systems, promote lipid redistribution because lipid molecules in these systems are inherently more dynamic. This facilitates the integration of hydrophobic anchors such as cholesterol or tocopherol moieties, which insert more readily when lipid packing favors their accommodation. In contrast, flat or rigid bilayers—particularly those enriched in cholesterol—tend to restrict lipid diffusion and resist reorganization, resulting in reduced insertion efficiency.^38^ Biotin–streptavidin anchoring also exhibits curvature dependence; in highly curved membranes the local biotin density may be reduced, diminishing the efficiency of streptavidin-mediated attachment, although careful adjustment of lipid composition can mitigate this effect. Beyond insertion, membrane configuration further modulates nanopore conductance states: planar bilayers show a higher probability of adopting high-conductance states at low voltages, whereas nanopipette-mounted membranes are more prone to enter low-conductance states, possibly due to lateral membrane pressure effects on pore stability.^15^ These findings highlight the importance of curvature-dependent optimizations when designing DNA nanopores for integration into vesicles, supported bilayers, or asymmetric membranes. It is likely that membrane curvature needs to be taken into account when nanopores are designed for integration into biological cells. Such designs must be adapted not only chemically to enable selective targeting of bacteria or eukaryotic cells, but also mechanically to accommodate their markedly different membrane properties.

## 4 Applications and Future Perspectives

Transmembrane-spanning DNA nanopores have rapidly transitioned from proof-of-concept nanostructures to functional components in a growing range of bionanotechnological applications. By providing controllable transmembrane access and programmable molecular interfaces, DNA nanopores enable functions that are difficult to achieve with conventional membrane systems, including selective molecular exchange, information transfer across membranes, and spatially confined biochemical reactions. As a result, they have been exploited in diverse application contexts, such as biosensing, controlled transport and release, and the construction of membrane-integrated synthetic and therapeutic systems. The following sections highlight representative applications of DNA nanopores and illustrate how their programmable nature translates into functional capabilities in complex biological and biomimetic environments.

### 4.1 Molecular Sensing

Beyond ion transport, recent work has demonstrated the ability of DNA nanopores to support single-molecule translocation and structural discrimination of nucleic acids based on the single-molecule resistive pulse sensing technique. As a first example,^16^ a barrel-shaped DNA nanopore inserted in a lipid membrane allowed the passage of ssDNA and its detection by the resistive pulse sensing technique, producing distinct ionic current signatures corresponding to individual translocation events. Although this system did not include recognition elements, it established the potential of DNA nanopores for electrical single-molecule detection.

Beyond biomolecular passage, more advanced single-molecule sensing applications have mainly been realized with DNA nanopores on solid-state supports, but it should, in principle, be possible to transfer the concepts to membrane-spanning DNA pores. DNA pores on solid-state supports have been equipped with complementary ssDNA probes in the nanopore lumen, allowing the pore to selectively bind and detect matching target sequences. Hybridization within the confined space of the pore led to prolonged and characteristic current blockades, enabling sequence-specific identification of nucleic acids (Figure 4b).^90^ Further refinements introduced aptamers into the pore interior to recognize secondary structures such as G-quadruplexes (G4). By attaching G4-specific aptamers, the nanopore could differentiate between folded and unfolded DNA based on blockade duration and depth, enabling structural analysis at the single-molecule level without labels.^92^ The site-specific inclusion of peptide- or oligo-based aptamers further offers a path to detect a broader spectrum of analytes including proteins, drugs, and biomarkers. These aptamers can be designed to extend from the pore lumen or surface, and their binding events can be monitored through resistive pulse sensing, potentially enhanced by conformational changes in the origami scaffold that amplify the signal-to-noise ratio. A recent study has shown that integrating DNA origami structures into solid-state nanopores can significantly enhance single-molecule detection sensitivity for proteins by amplifying ionic current blockades and dwell times.^93^ Such strategies, however, have so far not been implemented in membrane-inserted pores, but they represent an exciting direction for future development of functional biosensing platforms.

**Figure 4:**
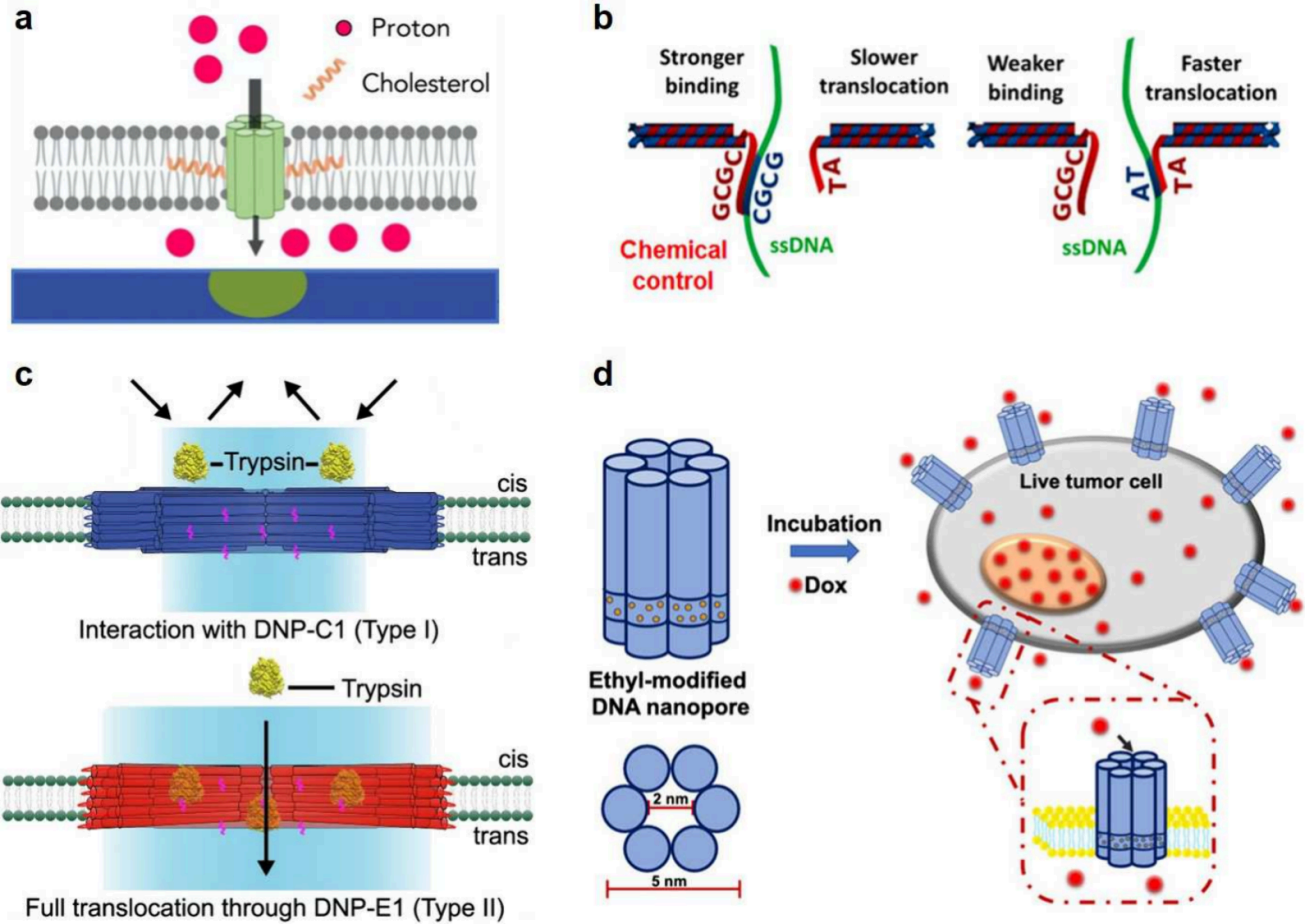
Applications and emerging directions of DNA nanopore technologies. (a) A cholesterol-functionalized DNA nanopore with two hydrophobic anchors facilitates transmembrane proton (H^+^) transport, mimicking biological ion channels. Reproduced with permission from ref 89. Copyright 2023 Luo et al. (b) DNA origami nanopores with sequence-specific binding sites enable selective detection of ssDNA based on nucleotide composition. Reproduced with permission from ref 90. Copyright 2013 American Chemical Society. (c) Schematic of triangular DNA nanopores undergoing dynamic shape changes between expanded and contracted states. In Type I events, trypsin transiently interacts with the contracted DNP-C1 conformation; in Type II events, trypsin fully translocates through the expanded DNP-E1 pore. Reproduced with permission from ref 32. Copyright 2024 The Authors. (d) A six-helix bundle DNA nanopore and its insertion in the plasma membrane of live cells. Reproduced from ref 91 under the terms of the Creative Commons Attribution License.

DNA structures can be further engineered with programmable vestibules or docking sites to control the timing and rate of translocation, laying the groundwork for highly tunable biosensors.^94^ This level of control opens the door to broader applications, including the detection of size- and structure-dependent features in a wide range of biomolecules. Potential targets include distinguishing between monomers and higher-order aggregates in protein misfolding diseases, probing the folding intermediates or tertiary motifs of functional RNAs, and monitoring enzymatic depolymerization of complex polysaccharides such as heparin or cellulose. For example, Thomsen et al.^12^ demonstrated a large DNA nanopore equipped with strand-lock controlled flaps, where toehold-mediated unlocking exposes hydrophobic anchors and enables membrane insertion in a programmable manner, providing a blueprint for externally triggered, condition-responsive biosensing platforms. Larger DNA nanopore designs have been developed to mimic nuclear pore complexes (NPCs)—macromolecular assemblies that regulate bidirectional transport between the nucleus and cytoplasm.^95^ These synthetic structures allow precise control over the type and copy number of incorporated nucleoporins (Nups), suggesting that they can support biologically relevant macromolecular transport.

In parallel, DNA nanopores have been engineered with tunable pore geometries, which directly impacts their ability to discriminate biomolecules by size, conformation, or interaction strength. Building on this structural programmability, Dey et al.^18^ reported a membrane-inserted DNA nanopore featuring a reversible nanomechanical lid, whose opening and closing could be precisely controlled via strand-displacement reactions, thereby enabling switchable gating and size-selective transport of macromolecular cargo across lipid bilayers. Complementary approaches have achieved geometry-responsive transport through lumen reconfiguration: a triangular DNA nanopore with adjustable DNA locks allowed real-time control over pore opening and cargo release in response to strand displacement or temperature cues (Figure 4c).^32^ Temperature-responsive gating has also been demonstrated by Arnott and Howorka,^96^ who exploited thermally induced structural rearrangements in a six-duplex nanopore to reversibly modulate pore accessibility and molecular transport. Notably, these systems demonstrated pronounced state-dependent molecular selectivity: the same cargo molecule, such as trypsin or fluorescent dyes, was strictly permitted or excluded depending on the conformational state of the pore, illustrating a functional mechanism for temporal molecular sorting and selective release. This design paradigm highlights the potential of DNA nanopores to function as dynamic transport modules, enabling molecular discrimination, signal-responsive gating, and targeted delivery in synthetic cells or therapeutic systems.

Beyond the above-mentioned advances, ongoing developments continue to introduce stimuli-responsive elements, transforming static pores into active, controllable gates. This functionality can be engineered by incorporating molecular switches that respond to voltage, light, pH, and ions. For instance, Seifert et al.^15^ demonstrated a bilayer-spanning DNA nanopore that could reversibly switch between open and closed conductive states in response to applied voltage, thereby regulating ionic transport across lipid membranes. The structure consisted of a barrel-shaped DNA origami channel with a controllable flap, mimicking the voltage-gated behavior of natural ion channels. This approach demonstrated not only the feasibility of electrically actuated gating in synthetic nanopores but also opened up possibilities for constructing responsive membrane devices with dynamic control over transport.

Offenbartl-Stiegert et al.^97^ demonstrated a membrane-inserted DNA nanopore featuring reversible light-gated opening and closing, achieved by incorporating photoresponsive azobenzene-modified DNA strands that undergo conformational switching upon illumination. This design enabled precise, reversible control over pore conductance using external light stimuli, highlighting the potential of optically addressable DNA nanopores for spatiotemporally controlled transport and biosensing.

Chen et al.^98^ reported a dynamic pH-responsive DNA cube nanopore in which pH-sensitive DNA motifs, specifically semi i-motif sequences, were strategically integrated into the structural elements of a DNA nanocube that inserts into lipid bilayers via cholesterol anchors. Upon a decrease in environmental pH, protonation of cytosine residues promotes the formation of semi i-motif structures, leading to conformational rearrangements that partially obstruct or constrict the nanopore lumen. At near-neutral pH, deprotonation destabilizes the semi i-motif, restoring a more open pore geometry and enhancing molecular transport. Through this reversible, protonation-driven structural transition, the nanopore exhibits adaptive transmembrane transport behavior that dynamically responds to environmental pH, exemplifying a programmable, stimuli-responsive channel design for artificial membrane systems.

An example of ion-responsive DNA origami was reported by Praetorius et al.^99^ In the absence of Zn^2+^ ions, the DNA origami architecture remained in a mechanically constrained state, in which predesigned locking strands and connecting elements held flaps or lids in a closed or inactive conformation, maintaining the nanopore in a blocked or restricted state and preventing transport or downstream reactions. Upon the introduction of Zn^2+^ ions, cleavage of the locking or connecting strands is triggered. Rupture of locking strands releases hinged elements, allowing flaps or lids to open, cleavage of connector strands relaxes structural tension and enables conformational reorganization, and removal of blocking elements clears the pore lumen, collectively resulting in an open and functional channel.

In addition to structural gating, chemical functionalization of the pore interior may further enable selective control of ionic transport properties. The native negative charge of DNA itself already favors cation selectivity, and this can be fine-tuned by attaching cation-coordinating motifs such as G4 (for K^+^) or peptide domains like zinc fingers (Zn^2+^) and calmodulin (Ca^2+^) or aptamers. These functional sites allow for metal ion sensing and gating, bringing DNA nanopores closer to the complexity of natural channels. Hybrid approaches are also under exploration. The incorporation of plasmonic nanoparticles (e.g., gold) at defined sites on DNA origami nanopores has been demonstrated to enable surface-enhanced Raman scattering (SERS).^100^ This could pave the way for dual-mode sensing systems that combine electrical and optical readouts. The feasibility of arraying DNA nanopores in parallel and encoding them with distinct recognition identifiers also raises the potential for multiplexed target detection. Such systems are highly relevant for next-generation sequencing and diagnostic applications, where simultaneous identification of different DNA, RNA, or protein biomarkers is required.

### 4.2 Cellular Applications

DNA nanostructures have emerged as a versatile class of synthetic compounds for cellular applications. They promise diverse strategies for targeted drug delivery, molecular sensing, and therapeutic diagnostics, often relying on the integration of molecular recognition elements and membrane-interacting motifs.^101,102^ Within this broader landscape, DNA nanopores constitute a distinctive subclass of DNA nanostructures, as they uniquely integrate programmable pore-forming architectures with cell-specific targeting and controlled transmembrane transport.^103^ Their modular design enables conditional membrane insertion and the formation of well-defined channels for ions or molecular cargo.

DNA nanopores functionalized with selective aptamers have been explored to achieve receptor-mediated insertion into specific cell membranes, such as those of cancer cells. Following membrane insertion, these nanopores act as conduits for drug molecules or ions, enabling localized delivery while minimizing off-target effects. A representative example is an adenine-binding fluorescent DNA aptamer functionalized with a hydrophobic tocopherol moiety,^74^ allowing spontaneous insertion of the aptamer into the outer leaflet of the plasma membrane without chemical modification of membrane proteins. Although not a transmembrane pore, the construct achieved stable localization on the cell surface and allowed real-time fluorescence imaging of extracellular ATP release from living astrocytes, revealing spatiotemporally resolved gliotransmitter dynamics synchronized with intracellular calcium wave propagation. This highlights how aptamer-equipped DNA nanostructures can support highly selective molecular recognition and chemical sensing at biological interfaces.

Related approaches have extended DNA nanopores toward direct membrane modulation and transport in living cells. Chemically modified membrane-spanning DNA nanopores bearing hydrophobic phosphorothioate belts have been shown to stably insert into cellular membranes, enabling ion or small-molecule transport and, in some cases, inducing cytotoxic effects through membrane permeabilization.^104,105^ More recent work demonstrated that enhanced hydrophobic modification strategies can significantly prolong nanopore residence on live cell membranes (Figure 4d), thereby improving transport efficiency and expanding the applicability of DNA nanopores in cellular delivery and therapeutic modulation.^91^

In parallel, DNA nanopores have also been explored as functional elements in synthetic vesicle and artificial cell systems. Large DNA origami nanopores capable of inserting into giant unilamellar vesicles have been developed to facilitate the passage of otherwise impermeable hydrophilic cargo, enabling controlled communication between vesicle interiors and their surroundings.^106^ Such designs provide a versatile platform for constructing drug-loaded vesicles or protocell systems in which molecular exchange is regulated by programmable DNA pores.

Beyond serving as static conduits, DNA nanopores can also be used in stimuli-responsive nanocarriers for drug delivery. In this scenario, a therapeutic payload is encapsulated within a lipid or polymer vesicle whereby release is governed by a DNA nanopore embedded in its membrane. The nanopore acts as a gated channel, and its opening or closing can be triggered by environmental cues such as pH, temperature, light, or specific molecular signals. Targeting moieties on the outer face of the nanopore can promote receptor-mediated cellular uptake, ensuring that drug release occurs only upon delivery to the desired cell type or tissue.

Despite significant advances in rational design and biological interfacing, several challenges remain before DNA nanopores can be translated into clinical applications.^107^ A major unresolved issue is the efficient targeting of DNA nanopores to intracellular membranes beyond the plasma membrane, such as endosomal, mitochondrial, or nuclear membranes. One promising future direction is the use of genetically encoded or cotranscriptional DNA/RNA origami, which enables endogenous production of nanostructures directly within cells. In addition, biostability and immune interactions remain critical concerns. DNA nanostructures are susceptible to nuclease degradation and may trigger innate immune responses depending on their sequence composition, size, and surface chemistry. Strategies such as chemical backbone modification, protective coatings, and dynamic degradation control have been proposed to balance structural stability with safe clearance from the body.

Taken together, these design considerations, including cell specificity, membrane anchoring, stimulus responsiveness, and intracellular trafficking control, position DNA origami nanopores as a powerful and adaptable platform for next-generation therapeutic systems. Ongoing research will continue to refine their pharmacokinetics, biodistribution, immune compatibility, and scalable manufacturing to enable real-world clinical translation.

### 4.3 Synthetic Biology

DNA nanopores offer a programmable platform for engineering synthetic signaling modules that mimic and interface with natural ion channels. Foundational work using tile-based DNA assemblies demonstrated that such structures can support voltage-responsive ionic currents, exhibit gating-like switching behavior, and induce membrane deformation, confirming their structural and functional competence as synthetic ion channels.^35^ Moreover, DNA nanopores have been shown to actively modulate membrane lipid composition and physical state, mimicking natural signaling initiation processes.^86^ This positions them as programmable tools for controlling synthetic membrane dynamics, with the potential to influence or even trigger cell fate decisions. Building on this, recent studies have advanced DNA nanopores into functional components of synthetic signaling modules. For instance, cholesterol-modified DNA nanopores have been integrated with bioprotonic devices to enable proton conduction across supported lipid bilayers, allowing real-time electronic readout of molecular binding events such as streptavidin or cardiac biomarker B-type natriuretic peptide recognition, without the need for chemical labeling.^89^ Furthermore, the coupling of DNA nanopores with field-effect transistors (FETs) has enabled the development of ion-sensitive devices capable of transducing ionic fluxes into electronic signals, paving the way for intracellular biosensing at the bioelectronic interface.^19^ These advances demonstrate the potential of DNA nanopores as modular components in synthetic signaling systems, where they can function as tunable conduits for chemical-to-electrical signal conversion, molecular logic gating, or programmable cellular communication interfaces.

Beyond their role as signal-transducing elements, these programmable pores also provide a general route to endow artificial membranes with controllable permeability and communication with their environment. In this sense, nucleic acid nanopores can not only be components of synthetic signaling circuits but also enabling modules for bottom-up synthetic biology, where membrane transport is a prerequisite for building cell-like systems.

In bottom-up synthetic biology, the ultimate aim is to construct a fully functional synthetic cell from first principles. This ambitious goal is being pursued through a stepwise approach, in which individual essential functions are implemented one after another. A variety of molecular design strategies can be employed toward this end, among which nucleic acid origami has emerged as a particularly versatile tool owing to its programmability and nanoscale precision. Here, we focus on one capability that is critical for a minimal synthetic cell: the formation of transmembrane pores. We then present some functions that such pores can fulfill in simple synthetic cell systems.

All living cells are enclosed by a membrane that serves essential roles in protection, compartmentalization, and metabolic regulation. However, this barrier is inherently impermeable to many biologically relevant molecules, making the selective transport of nutrients and waste products an essential function.^108^ In existing biology, this function is largely mediated by protein pores and transporters.^109^ In synthetic biology, nucleic acid origami pores have emerged as a useful alternative to protein-based channels, in particular for the import of larger macromolecules. It has been reported that synthetic DNA pores can support DNA-triggered and charge-selective release of small-molecule cargo from membrane-confined reservoirs, demonstrating their capability to actively regulate molecular transport in a manner analogous to gated protein channels.^110^

Recent advances demonstrate that DNA origami pores can enable synthetic cells with novel communication and signaling functions. Qiu et al.^111^ showed that DNA origami pores embedded in lipid membranes can mediate programmable communication between artificial cells. External DNA hairpin signals were transported into GUVs through the pores and enzymatically processed inside, generating linker strands that subsequently diffused out to trigger the aggregation of neighboring SUVs to the membrane. Importantly, the process was reversible, as complementary releaser strands could dissociate the aggregates. This established a synthetic system that both produces and receives biochemical signals and responds with coordinated collective behaviors. Complementarily, Jahnke et al.^112^ engineered DNA origami pores as signaling units that inserted into lipid membranes and functioned as nanopores capable of transporting DNA strands into GUVs. Once inside, these strands triggered the disassembly of encapsulated DNA filaments, which mimicked a cytoskeleton, thereby achieving chemical signal transduction. In addition, the pores were designed to directly bind the DNA filaments, such that clustering of the pores via biotin–streptavidin bridges at the membrane surface mechanically reorganizes the internal filament network, thereby establishing a synthetic form of signal transduction across the membrane.

Beyond nucleic-acid-encoded signals, chemical energy inputs have also been harnessed to drive responsive behavior in synthetic cell systems. Peng et al.^113^ developed a DNA-based artificial molecular signaling system in which ATP-responsive DNA nanogates integrated into vesicle membranes couple external ATP signals to internal reaction networks. ATP binding and hydrolysis triggered conformational switching of the nanogates, enabling controlled molecular transport and downstream signal processing inside the vesicles. This work demonstrates that DNA-based membrane pores and gates can interface chemical energy cues with programmable information processing, further expanding the repertoire of synthetic cell communication and response mechanisms. Together, these studies showcase how DNA origami pores can be applied in synthetic biology to implement synthetic functions, ranging from controlled cell–cell interactions to adaptive intracellular remodeling, thus providing a versatile platform for building responsive and communicative protocells. In parallel, hybrid DNA nanopore architectures, combining DNA origami with solid-state nanopores, have been developed to integrate the programmability of DNA nanostructures with the robustness and electrical readout of inorganic nanopores, extending their applicability to hybrid synthetic–bioelectronic systems and single-molecule sensing within synthetic cell frameworks.^114,115^

While DNA origami transmembrane nanopores have established themselves as powerful and programmable tools for creating complex membrane functions, cotranscriptional RNA origami holds particular promise for the next generation of bottom-up synthetic biology. RNA origami has several advantages: First, folding occurs cotranscriptionally during RNA synthesis by RNA polymerase, eliminating the need for temperature ramps or elaborate folding protocols.^116^ Second, replication, which would be difficult to implement with DNA origami, is inherent to RNA production, as many copies can be transcribed from a single DNA template. This natural coupling of genotype and phenotype not only simplifies synthesis but also provides a foundation for the implementation of directed evolution in the future. In addition, powerful design tools for cotranscriptional RNA origami now enable the rational design of functional structures, including membrane pores.^25,26^ At the same time, RNA origami still faces practical trade-offs relative to DNA origami. Cotranscriptional RNA origami structures are typically smaller: the largest cotranscriptionally folded design reported so far comprises ∼2500 nt,^25^ while annealed RNA assemblies can reach ∼6000 nt.^117^ This may make the design of more elaborate or larger pores—similar to DNA pores such as the T-shaped^11^ or funnel-shaped^17^ pores—challenging; these designs employed 7,560 nt- and 7,249 nt-long scaffolds, respectively.

Furthermore, whereas DNA origami readily supports modular substitution of individual staple strands (for example, chemically modified strands that facilitate membrane binding), RNA origami is typically encoded as a single strand; consequently, even small functional changes often require redesign of the full sequence and synthesis of a new DNA template. Nevertheless, membrane anchoring of RNA-based pores could be achieved through hybridization with chemically modified DNA or RNA oligomers (however, note that chemically modified RNA is more expensive than DNA as of this writing, typically about 3-fold more at common commercial vendors), or by directly encoding anchoring motifs. For example, aptamers such as the biotin aptamer have already been successfully integrated into RNA origami as membrane anchors.^68,69^ Here, however, a noteworthy challenge is that DNA templates typically cannot contain long repetitive regions,^118^ which limits repeated use of identical aptamers within a single structure. Taken together, these advances highlight RNA origami as a uniquely suited platform for creating self-producing, evolvable, and functional transmembrane structures in synthetic cells.

To date, only one study has demonstrated the potential of RNA as a building block for membrane pores. Li et al.^24^ reported the assembly of RNA nanotubes from multiple RNA strands. Although this approach did not employ cotranscriptional folding and thus lacks the replication and folding advantages outlined above, it showed that RNA can self-assemble into stable nanotube structures. Incorporation of cholesterol-modified strands enabled insertion of the pore into lipid bilayers and mediation of ionic conductance, underscoring RNA’s potential as a building block for functional synthetic cell hardware, such as membrane pores.

### 4.4 Challenges for Applications

Despite the programmable nature of nucleic acid nanopores, several bottlenecks remain to be addressed for their robust application.

#### 4.4.1 Leakless Transport

A persistent challenge for DNA/RNA nanopores in sensing as well as synthetic biology and medicine is the occurrence of ion leakage around the pore’s perimeter in addition to conductance through its central lumen. This is due to the formation of a “toroidal” lipid pore where the membrane bends to hide its hydrophobic core from the hydrophilic nucleic acid backbone.^119^ Potential solutions: Strategies to mitigate this include using charge-neutral chemical modifications, such as PPT groups,^14^ to mask the negative DNA backbone and promote “leakless” insertion without substantial lipid rearrangement.

#### 4.4.2 Selectivity and Molecular Discrimination

While small pores (∼2 nm) exhibit some degree of cation selectivity due to the negative charge of the DNA helices,^120^ achieving the high specificity required for complex biological function remains difficult. Potential solutions: Selectivity can be enhanced by making use of ion-sensitive DNA/RNA motifs^121^ or incorporating “smart” gating mechanisms, such as toehold-mediated strand displacement flaps^122^ or internal functionalization with aptamers and ligands to create specific molecular recognition sites. For ion selectivity, the leakage problem has to be solved (see above).

#### 4.4.3 Structural Stability

DNA nanostructures often face stability issues in physiological environments due to low concentrations of divalent ions, elevated temperatures or the presence of nucleases.^123^ Potential solutions: Stability can be improved by employing covalent cross-linking,^124^ using protective coatings^125^ like oligolysines,^126^ silanization,^127^ non-natural nucleic acids like XNA^128^ or mirror forms.^129^ In all cases, it has to be ensured that these interventions remain compatible with membrane insertion.

#### 4.4.4 Preventing Cellular Uptake for Long-Term Insertion

For therapeutic or synthetic biology applications, maintaining the nanopore on the cell surface is critical. However, cells naturally internalize foreign nanostructures via endocytosis,^130^ which limits the lifetime of the functional pore on the membrane. Potential solutions: To favor long-term insertion over uptake, pores can be designed with larger “cap” structures that physically hinder endocytic curvature or by functionalizing the pores with specific anchors that tether them to the extracellular matrix or membrane proteins.^131^

#### 4.4.5 Understanding Insertion into Cellular Membranes

There is currently a lack of complete understanding regarding the insertion of nucleic acid pores into cellular membranes where membrane proteins and the glycocalyx may present cell-type dependent physical barriers. Moreover, the composition of cellular membranes is much more complex than the model membrane systems used in most studies. Potential solutions: Systematic cell insertion studies are required for different combinations of cell types and pore designs. Targeted anchoring using receptor–ligand interactions or covalent tagging^132^ should also be explored beyond hydrophobic anchors.

#### 4.4.6 Immunogenicity

In vivo application of large DNA/RNA structures can trigger the innate immune system via Toll-like receptors (TLRs) that recognize foreign nucleic acids.^133^ Potential solutions: Depending on the application, it will be key to prevent uptake and/or to tune the innate response as required. This can be done by including innate triggers, like CPGs,^134^ or silencers, like chemical modification, use of unnatural structural features etc. Additionally, encapsulating these pores within biocompatible delivery vehicles until they reach the target membrane may reduce systemic exposure.

## 5 Conclusion

The development of nucleic acid origami nanopores represents a significant advancement in nanoscale engineering, providing a versatile platform for applications ranging from biosensing and molecular transport to synthetic biology and targeted drug delivery. By leveraging the programmability of DNA self-assembly, researchers have designed nanopores with highly tunable structural properties, including size, geometry, functionalization, and membrane integration strategies. The ability to precisely control ionic conductance pathways through these nanopores has enabled their use in single-molecule sensing, ion transport studies, and molecular discrimination. However, challenges such as insertion efficiency, lipid rearrangement effects, and conductance variability must be carefully managed to optimize nanopore performance in biological and synthetic membrane systems. Strategies including hydrophobic functionalization, biotin–streptavidin anchoring, spacer optimization, and membrane curvature engineering have been explored to enhance nanopore stability, ensure efficient membrane integration, and regulate ionic transport dynamics. Future research in nucleic acid nanopore technology should focus on refining membrane interaction mechanisms to further improve conductance stability and selectivity. Advances in hybrid DNA–protein nanopores, stimuli-responsive gating mechanisms, and dynamic lipid membrane engineering could enable more sophisticated and adaptive nanopore functions. Additionally, integrating DNA nanopores with portable biosensing devices and high-throughput analytical platforms will accelerate their transition from fundamental research to real-world applications in medical diagnostics, environmental monitoring, and targeted therapeutics. An exciting future direction could be genetically encodable RNA origami pores. By addressing current limitations and harnessing new design principles, nucleic acid nanopores will continue to evolve as powerful tools for studying and manipulating biological systems at the nanoscale.
